# Operative efficiency: a comparative analysis of Versius and da Vinci robotic systems in abdominal surgery

**DOI:** 10.1007/s11701-023-01806-5

**Published:** 2024-03-22

**Authors:** Mouhammad Halabi, Kayanne Khoury, Abdulrahman Alomar, Joseph El Dahdah, Obai Hassan, Khadija Hayyan, Engy Bishara, Hatem Moussa

**Affiliations:** 1 Department of Surgery, American Hospital Dubai, Dubai, United Arab Emirates; 2 School of Medicine, Royal College of Ireland –Bahrain, Busaiteen, Bahrain; 3 Department of Cardiovascular Medicine, Heart and Vascular Institute, Cleveland Clinic Foundation, Cleveland, OH USA

**Keywords:** Robotic surgery, Versius, da Vinci, Cholecystectomy, Hernia

## Abstract

Robotic-assisted surgery has gained momentum in the pursuit of improved minimally invasive procedures. The adoption of new robotic platforms, such as the Versius, raises concerns about safety, efficacy, and learning curves. This study compares the Versius to the well-established da Vinci in terms of operative time and patient population. Retrospective data collection was conducted on patient data from inguinal hernia surgery, ventral hernia surgery, and cholecystectomies performed between February 2022 and March 2023 at the American Hospital of Dubai. Only experienced cases were included, ensuring proficiency with robotic technology. Versius had longer procedure times in inguinal and ventral hernia surgeries but not in cholecystectomy. No intraoperative complications were observed in either system. This study demonstrates that Versius can provide comparable outcomes to the da Vinci in abdominal surgery, with no observed intraoperative complications.

## Introduction

Over the years, robot-assisted surgery’s potential to deliver better outcomes in minimally invasive surgery (MIS) has gained considerable attention. Slowly straying from traditional and conventional surgical methods, healthcare centers worldwide have begun adopting this transformative approach with the aim of delivering better post-operative outcomes as well as minimizing recovery time [1].

When introducing a new robotic platform into a hospital system, prioritizing patient safety is paramount. Given the swift adoption of this technology by hospitals worldwide, it is crucial to exercise caution in order to minimize the risk of adverse events.

Introducing a novel robotic system with comparable developmental milestones to its predecessors, the CMR Versius features individual robotic arms mounted on separate carts, eliminating the need for docking to the trocars. The company places a strong emphasis on addressing issues of cost-effectiveness, seamless connectivity, and simplified software updates, aligning with a biomedical engineering approach [2].

Although previous studies have discussed the implementation of the Versius robotic surgery platform for abdominal surgery [3–6], none have compared its performance to that of another robotic system, such as the da Vinci, a relatively well-established robot that has pioneered the domain of robot-assisted surgery. It is imperative to do so in order to assess the feasibility of using the Versius to deliver safe and reproducible outcomes.

This study describes the multifaceted process of implementing the Versius surgical robotic system at an experienced robotic surgery center. Herein, we present the data regarding the performance of the Versius in inguinal hernia, ventral hernia, and cholecystectomy procedures across both demographic and clinical parameters.

## Methods

This study retrospectively reviewed maintained data for patients who underwent hernia and gallbladder surgery between February 2022 and March 2023 at the American Hospital of Dubai (AHD). The robotic platforms used were the da Vinci Robot (Xi–OS4; v9, Intuitive Surgical, Inc., Sunnyvale, California) and the CMR Versius (Cambridge, United Kingdom). The study was approved by the AHD Institutional Review Board.

The data included in this study encompasses experienced cases only (an experienced case was defined after a surgeon has surpassed the learning curve, which was after 15 cases) that were performed by a single experienced surgeon, which has performed 700 procedures utilizing the da Vinci system and 230 procedures using the Versius system. Thus, only data collected following the completion of comprehensive training and certification were included in this study. This selective approach ensured that outcomes Reflected the surgeon’s proficiency with the robotic technology. Cases at the beginning of the learning curve (<15 cases) were excluded from this study.

The following variables were analyzed: age, sex, body mass index (BMI), and past medical history (which included comorbidities, such as hypertension, diabetes mellitus, dyslipidemia, and cardiovascular disease), previous surgeries, and total operative time. The total operative time for any robotic procedure was defined as console start to console finish; no docking times were included. Additionally, for analytical clarity, comorbidities were quantified as a unified category denoted by "1" without specific individual specifications.

All patients in this study were required to be over the age of 18 and had to have undergone either cholecystectomy, inguinal hernia repair, or ventral hernia repair between February 2022 and March 2023. Any patients that did not fit these criteria were excluded from this study.

Data are presented as mean ± standard deviation. Analysis of 249 patients was performed using the student’s *t *test for means and chi-square analysis for categorical variables. Significance was defined as *P* < 0.05.

## Results

Table 1 summarizes demographic, clinical, and procedural data regarding inguinal hernia surgery performed with either the Versius or the da Vinci. The mean age for patients undergoing surgery with the Verisus was 44 years old (±11.3 SD), while that of da Vinci patients was 46.2 years old (±12.1 SD), with no significant difference observed (*P* = 0.29). In terms of gender distribution, 6% of patients that underwent surgery using the Versius robot were female, while 11.5% of the patients that underwent surgery with the da Vinci were female (*P* = 0.234).

**Table 1:** Inguinal hernia

	Inguinal Hernia		
	Versius (*N* = 50)	da Vinci (*N* = 78)	*P* value
Demographics			
Age in years, mean (±SD)	44 (11.3)	46.2 (12.1)	*0.29*
Females, *N* (%)	3 (6)	9 (11.5)	*0.234*
Clinical characteristics			
BMI in kg/m^2^, mean (±SD)	25.6 (3.1)	27.1 (4.2)	*0.023*
Alcohol use, *N* (%)	12 (24)	8 (10.3)	*0.03*
Tobacco use, *N* (%)	7 (14)	5 (6.4)	*0.123*
Hypertension, *N* (%)	0	11 (14.1)	*0.003*
Diabetes Mellitus, *N* (%)	3 (6)	7 (9)	*0.384*
Dyslipidemia, *N* (%)	10 (20)	10 (12.8)	*0.199*
History of Cardiovascular disease, *N* (%)	3 (6)	5 (6.4)	*0.618*
History of Abdominal Surgery, *N* (%)	16 (32)	30 (38.5)	*0.319*
Number of comorbidities, *N* (%):			*0.07*
*0*	17 (34)	42 (53.8)	
*1*	15 (30)	22 (28.2)	
*2*	9 (18)	9 (11.5)	
*3*	7 (14)	5 (6.4)	
*4*	2 (4)	0	
Procedure length in minutes, mean (±SD)	103 (28.5)	72.3 (28.3)	< *0.001*

BMI was significantly different between both groups (*P* = 0.023), with the mean BMI of the Versius patient group being 25.6 kg/m^2^ (±3.1 SD) and the da Vinci group being 27.1 kg/m^2^ (±4.2 SD). Alcohol use was significantly greater (*P* = 0.03) in the Versius group (24%) when compared to the da Vinci group (10.3%). Cases of hypertension were significantly greater (*P* = 0.003) in the da Vinci group (14.1%) when compared to the Versius group (0%). Overall, there was no significant difference (*P* = 0.07) in the number of comorbidities between both groups. The mean procedure length in the Versius group was significantly longer than that of the da Vinci group (103 ± 28.5 vs. 72.3 ± 28.3 SD mins respectively, *P* < 0.001).

Table 2 presents data on ventral hernia surgery. There was no significant difference in the mean age of patients who underwent surgery using the Versius and those using da Vinci (*P* = 0.164) at 37.1 years old (±8.1 SD) and 42.6 years old (±14.5 SD), respectively. The gender distribution was significantly different between both groups (*P* = 0.009), with females making up 75% of patients that underwent surgery using the Versius as compared to 30% using da Vinci.

**Table 2:** Ventral hernia

	Ventral Hernia		
	Versius (*N* = 16)	da Vinci (*N* = 20)	*P* value
Demographics			
Age in years, mean (±SD)	37.1 (8.1)	42.6 (14.5)	0.164
Females, *N* (%)	12 (75)	6 (30)	0.009
Clinical characteristics			
BMI in kg/m^2^, mean (±SD)	28.9 (3.9)	29.6 (5.4)	0.656
Alcohol use, *N* (%)	1 (6.3)	6 (30)	0.084
Tobacco use, *N* (%)	4 (25)	5 (25)	0.647
Hypertension, *N* (%)	0	7 (35)	0.009
Diabetes Mellitus, *N* (%)	0	2 (10)	0.302
Dyslipidemia, *N* (%)	3 (18.8)	4 (20)	0.631
History of Cardiovascular disease, *N* (%)	1 (6.3)	1 (5)	0.698
History of Abdominal Surgery, *N* (%)	6 (37.5)	11 (55)	0.194
Number of comorbidities, *N* (%):			0.118
*0*	5 (31.3)	9 (45)	
*1*	7 (43.8)	2 (10)	
*2*	3 (18.8)	3 (15)	
*3*	1 (6.3)	4 (20)	
*4*	0	2 (10)	
Procedure length in minutes, mean (±SD)	135 (46.1)	94.1 (36.5)	0.007

No significant differences in social history were observed between both groups. Cases of hypertension were significantly greater (*P* = 0.009) in the da Vinci group (35%) when compared to the Versius group (0%). History of abdominal surgery was noted in 37.5% of patients in the Versius group compared to 55% in the da Vinci group, demonstrating no significant difference (*P* = 0.194). Altogether, there was no significant difference in the number of comorbidities between both groups (*P* = 0.118). The mean procedure length was significantly longer in the Versius group when compared to the da Vinci group (135 ± 46.1 vs. 94.1 ± 36.5 mins, *P* = 0.007).

Table 3 outlines the data on cholecystectomy procedures. The mean age of patients in the Versius group was 44.3 years old (±13.6 SD), while that of the da Vinci group was 44.3 years old (±10.4 SD), with no significant difference (*P* = 0.987). Females comprised 59.3% of patients in the Versius group, which is insignificantly different when compared to the 64.5% comprising the da Vinci group (*P* = 0.404). There was no significant difference observed between the BMI in both groups (*P* = 0.661), with the mean BMI in the Versius group being 29.0 kg/m^2^ (±4.3 SD) and the da Vinci group being 29.52 kg/m^2^ (±6.3 SD). Cases of hypertension were significantly greater (*P* = 0.002) in the da Vinci group (19.4%) as compared to those in the Versius group (0%). Patients with diabetes mellitus in the Versius group were recorded at 11.1%, while those in the da Vinci group were at 22.6%, indicating no significant difference (*P* = 0.144). A history of abdominal surgery was noted in 38.9% of patients in the Versius group as opposed to 35.5% in the da Vinci group, with no significant difference observed (*P* = 0.392). Differences in patients’ overall number of comorbidities were insignificant between both groups (*P* = 0.793).

**Table 3:** Cholecystectomy

	Cholecystectomy		
	Versius (*N* = 54)	da Vinci (*N* = 31)	*P* value
Demographics			
Age in years, mean (±SD)	44.3 (13.6)	44.3 (10.4)	0.987
Females, *N* (%)	32 (59.3)	20 (64.5)	0.404
Clinical characteristics			
BMI in kg/m^2^, mean (±SD)	29.0 (4.3)	29.52 (6.3)	0.661
Alcohol use, *N* (%)	3 (5.6)	2 (6.5)	0.63
Tobacco use, *N* (%)	5 (9.3)	3 (9.7)	0.65
Hypertension, *N* (%)	0	6 (19.4)	0.002
Diabetes Mellitus, *N* (%)	6 (11.1)	7 (22.6)	0.144
Dyslipidemia, *N* (%)	9 (16.7)	2 (6.5)	0.148
History of Cardiovascular disease, *N* (%)	4 (7.4)	1 (3.2)	0.387
History of Abdominal Surgery, *N* (%)	21 (38.9)	11 (35.5)	0.392
Number of comorbidities, *N* (%):			0.793
*0*	18 (33.3)	10 (32.3)	
*1*	21 (38.9)	10 (32.3)	
*2*	9 (16.7)	6 (19.4)	
*3*	5 (9.3)	5 (16.1)	
*4*	1 (1.9)	0	
Procedure length in minutes, mean (±SD)	72.7 (23.2)	82.6 (24.1)	0.079

In contrast to both inguinal hernia and ventral hernia surgeries, no significant difference was observed in the procedure length between the Versius and the da Vinci (72.7 ± 23.2 vs. 82.6 ± 24.1 mins respectively, *P* = 0.079).

Table 4 condenses the data on all surgeries. There was no significant difference observed in the mean age of patients (*P* = 0.204) in both the Versius group and the da Vinci group, with values recorded at 43.23 years old (±12.21 SD) and 45.19 years old (±12.1 SD), respectively. The gender distribution consisted of 39.2% of patients being female in the Versius group compared to 27.1% in the da Vinci group. BMI was insignificantly different (*P* = 0.381) with the mean BMI in the Versius group being 27.53 kg/m^2^ (±4.11 SD) and that of the da Vinci group being 28.04 kg/m^2.^ (±5.0 SD). Cases of hypertension were significantly greater (*P* < 0.001) in the da Vinci group (18.6%) when compared to those in the Versius group (0%). The difference in patients with a history of cardiovascular disease was not significant (*P* = 0.435) when comparing the Versius group (6.7%) to the da Vinci group (5.4%). History of abdominal surgery was noted in 35.8% of patients in the Versius group compared to 40.3% in the da Vinci group, demonstrating no significant difference (*P* = 0.331). Overall, there was no significant difference (*P* = 0.141) in the number of comorbidities between both patient groups.

**Table 4:** All procedures

	All surgeries		
	Versius (*N* = 120)	da Vinci (*N* = 129)	*P* value
Demographics			
Age in years, mean (±SD)	43.23 (12.21)	45.19 (12.1)	0.204
Females, *N* (%)	47 (39.2)	35 (27.1)	
Clinical characteristics			
BMI in kg/m^2^, mean (±SD)	27.53 (4.11)	28.04 (5.0)	0.381
Alcohol use, *N* (%)	16 (13.3)	16 (12.4)	0.446
Tobacco use, *N* (%)	16 (13.3)	13 (10.1)	0.242
Hypertension, *N* (%)	0 (0)	24 (18.6)	< 0.001
Diabetes Mellitus, *N* (%)	9 (7.5)	16 (12.4)	0.137
Dyslipidemia, *N* (%)	22 (18.3)	16 (12.4)	0.125
History of cardiovascular disease, *N* (%)	8 (6.7)	7 (5.4)	0.435
History of Abdominal Surgery, *N* (%)	43 (35.8)	52 ( 40.3)	0.331
Number of comorbidities, *N* (%):			0.141
*0*	40 (33.3)	61 (47.3)	
*1*	43 (35.8)	34 (26.4)	
*2*	21 (17.5)	18 (14)	
*3*	13 (10.8)	14 (10.9)	
*4*	3 (2.5)	2 (1.6)	

In this entire series, we observed no intraoperative complications in both the Versius and the da Vinci groups.

## Discussion

In this study, we conducted a comprehensive analysis of demographic, clinical characteristics, and procedure lengths for patients undergoing surgery with the Versius surgical system to validate the safety and determine whether the risks associated with obtaining a new robotic surgery platform are outweighed by its outcomes.

We were able to demonstrate that surgical procedures with the Versius can have comparable outcomes to that of the da Vinci in abdominal surgery. Furthermore, the learning curve on this new system was very minimal as all the procedures done were performed by experienced robotic surgeons, thus making the learning curve obsolete.

Patients undergoing surgery with the Versius shared similar demographics to those who underwent surgery with the da Vinci from our initial experience.

Although CMR’s Versius exhibits an array of positive characteristics in minimal access surgery—such as an ergonomic platform that allows for flexible placement of ports and versatility leading to enhanced proficiency—surgical systems, like any man-made machine, are subject to faults that may hinder certain outcomes.

A study on robotic oncologic colorectal surgery by Huscher et al. demonstrates that the high degree of Versius’s versatility is, among other factors, due to the fact that the robotic arms are independent of one another. Despite this, the independence of robotic arms is also a contributing factor to increased operative time, as they tend to clash together without coordinated surveillance. Docking was found to be time-consuming as well.

Versius is a novel robot that slightly differs from the standard of other robotic systems, leading to extra precautionary measures to be taken to decrease the fighting and clashing of instruments [7].

Also, the positions of bedside units (BSUs) were found to limit the assistant surgeon from having a proper position, which led to uncomfortable adjustments when performing high-risk tasks. Another point found within this study is that the platform’s dexterity was not optimal due to missing certain anatomical structures while adjusting the directions of the instruments [7]. However, the flexibility of the bedside units provided increased flexibility in port placement, which has reduced the learning curve, making the initial experience with such a system preferable [8–10].

In spite of capturing high-quality images due to 3D technology, Huscher et al. reports that improved systems, such as near-infrared imaging for fluorescence, hyperspectral imaging, and picture-in-picture visualization, are not available on the platform. In addition, Versius does not have specific instruments, such as coagulation devices, staplers, aspirators, advanced sealers, and clip appliers [6, 7].

In robotic-assisted visceral surgery, Wehrmann et al. found that the focal point of the platform is always set to one space, making it nearly impossible to change from one part of the abdomen to another e.g., the right upper quadrant to the right lower quadrant, leading to the necessity of utilizing laparoscopic steps alongside robotic [6].

In another study on robotic oncologic colorectal surgery, Collins et al. also found difficulties in attempts to simultaneously work in more than one abdominal quadrant. Collins et al. reports that external clashing of robotic arms occurred due to increased mobility of the colon and longer instrument paths. As previously mentioned, lack of specific instruments led to the need to adopt a hybrid approach of laparoscopic and robotics in specific patient populations e.g., obese patients, which was evident in Collins et al.’s experience. In the case of a lower anterior resection, laparoscopic mobilization of the splenic flexure was done to save time, while in an extended right hemicolectomy, robotic splenic flexure mobilization was performed initially, followed by completion laparoscopically [11].

When compared to the da Vinci, the Versius robotic surgery platform demonstrated significantly longer operating times in inguinal hernia repair and ventral hernia repair, however, no significant difference was seen in procedure length for cholecystectomy cases. The patient population used in this study was also comparable, with very few significant differences in comorbidities between the two.

Our study is retrospective in nature; as such it is only natural that we have important limitations that should be outlined. We had to keep our inclusion/exclusion criteria as broad as possible in order to obtain a reasonable number of patients for analysis. This may have introduced heterogeneity into our study which may have led to confounding. Considering our study was conducted at a single center, this limited our ability to generalize our results. Moreover, our sample size was restricted, which may have affected the statistical power and the reliability of our results.

To our knowledge, we have the highest volume of patients undergoing robotic surgery in the Middle East and North Africa region. The success of our program has allowed us to adopt both the Versius and the da Vinci robotic platforms, which allows us to serve as a center for comparative studies.

Although CMR has not proposed precautions to prevent clashing of robotic arms, altering the position of BSUs and calibrating them through a tailored approach to each patient, based on their anatomy and BMI, alongside monitoring of the equipment can prevent clashing. Since Versius is fundamentally based on end-user feedback, i.e., the surgical team, adjustments can be made by adding specific instruments that minimize the need for a hybrid approach. Updates can also be made to the platform’s interface by adding revolutionized visualization systems such as near-infrared imaging.

This study highlights that while the Versius system has its unique challenges, it can be a valuable addition for new robotic surgery programs and offers potential benefits that can be harnessed with the right adaptations and movements.

## Conclusion

This study compared the initial outcomes of the Versius and da Vinci robotic systems in abdominal surgery. Both systems were free of intraoperative complications, revealing relatively safe outcomes when comparing the Versius to the da Vinci.

At the moment, the main limitations of the Versius seem to be instrument availability as well as its fixed focal point. While the Versius does offer remarkable advantages in terms of flexibility and versatility, further research is needed to optimize its performance and address its limitations, thus emphasizing the need for continued advancements in the world of robotic surgery.

## Data Availability

The data presented in this study are available on request from the corresponding author. The data are not publicly available due to privacy and ethical reasons.
